# Retraction Note: Risk factors, clinical presentations and predictors of stroke among adult patients admitted to stroke unit of Jimma university medical center, south west Ethiopia: prospective observational study

**DOI:** 10.1186/s12883-019-1564-3

**Published:** 2019-12-17

**Authors:** Ginenus Fekadu, Legese Chelkeba, Ayantu Kebede

**Affiliations:** 1 Clinical Pharmacy Unit, Department of Pharmacy, Institute of Health Sciences, Wollega University, P.O Box 395, Nekemte, Ethiopia; 2 School of Pharmacy, Institute of Health, Jimma University, Jimma, Ethiopia; 3 Department of Epidemiology, Institute of Health, Jimma University, Jimma, Ethiopia

**Retraction Note: BMC Neurol (2019) 19:183**

**https://doi.org/10.1186/s12883-019-1412-5**

The Editor and Publisher have retracted this article [1]. This article was published as the result of a technical error which resulted in two versions [1, 2] of the same article being published. [2] is the final version of the article. Springer Nature apologises to the authors and to readers for the inconvenience caused. All authors agree with this retraction.

## References

[1] Fekadu G, Chelkeba L, Kebede A (2019). Risk factors, clinical presentations and predictors of stroke among adult patients admitted to stroke unit of Jimma university medical center, south west Ethiopia: prospective observational study. BMC Neurol.

[2] Fekadu G, Chelkeba L, Kebede A (2019). Risk factors, clinical presentations and predictors of stroke among adult patients admitted to stroke unit of Jimma university medical center, south west Ethiopia: prospective observational study. BMC Neurol.

